# Causes of Melena and Effective Examination Strategies in Children

**DOI:** 10.3389/fped.2021.780356

**Published:** 2021-12-08

**Authors:** Itaru Iwama, Masashi Yoshida, Tomoko Hara, Ryusuke Nambu

**Affiliations:** Division of Gastroenterology and Hepatology, Saitama Children's Medical Center, Saitama, Japan

**Keywords:** melena, children, esophagogastroduodenoscopy, capsule endoscope, duodenal ulcer

## Abstract

**Background and Aim:** Melena, or tarry black stool, is not a rare symptom encountered in pediatric clinical practice, and the bleeding source varies from the upper gastrointestinal tract to the small intestine. Endoscopy is effective in identifying bleeding, but it does not always identify the source of bleeding. Endoscopic examination in children is commonly challenging, and there are no detailed reports about the causes of melena in children. This observational study aimed to validate the cause of melena in children and to investigate more effective and less burdensome examination methods.

**Methods:** We retrospectively reviewed the clinical records of 55 patients who underwent examination for melena.

**Results:** In this research, 38 patients had underlying diseases such as malignancy and severe mental and physical disorders. The bleeding source was identified in 39 patients. The most common final diagnosis was duodenal ulcer (*n* = 22), and the other diagnoses were gastric ulcer, esophagitis, and esophageal varices. The upper gastrointestinal tract was the most common source of bleeding (*n* = 34). In five patients, the bleeding source was the small intestine. Vomiting, abnormal abdominal ultrasonography findings, and a hemoglobin level of ≤ 3 g/dL than the lower normal limit were significant factors indicating that the bleeding source can be found on esophagogastroduodenoscopy.

**Conclusions:** The upper gastrointestinal tract was the most common bleeding source of melena in children. As in adults, esophagogastroduodenoscopy is the primary endoscopic method of choice. Furthermore, small bowel capsule endoscopy may be useful in identifying the bleeding source in children without upper gastrointestinal lesions.

## Introduction

Melena, or tarry black stool, is not a rare symptom in pediatric clinical practice, and the source of hemorrhage varies from the upper gastrointestinal tract to the small intestine. In rare cases, fatal bleeding may occur, thereby requiring the prompt identification and treatment of the bleeding source (1). Endoscopy is effective in identifying the bleeding source, and small bowel capsule endoscopy (SBCE) and balloon-assisted enteroscopy (BAE) have been used to detect small intestinal diseases that were previously considered as obscure gastrointestinal bleeding (2). Gastrointestinal endoscopy is also useful but sometimes challenging to perform in children. The endoscopes that can be used for small infants are limited, and sedation is essential for safe examination. There have been several reports about upper gastrointestinal bleeding in children (3–5). However, the source of bleeding and the disease of melena or tarry black stools have not been reported. Although diagnostic algorithm for gastrointestinal bleeding in adults has been established (6), it cannot be applied to children with different causative diseases. Thus, a pediatric-specific algorithm is required. Therefore, this observational study aimed to assess the source and cause of melena in children and a more effective and less burdensome examination method.

## Methods

### Patients

Patients who were admitted for an examination of melena at Saitama Children's Medical Center between April 2016 and June 2021 and those who presented with melena during hospitalization were included in this study. Melena was defined as the presence of black stool as claimed by family members and as confirmed via stool examination performed by a pediatric gastroenterologist. The following information was collected retrospectively from the medical records: age, sex, underlying disease, accompanying symptoms, hemoglobin (Hb) and hematocrit (Ht) levels, blood urea nitrogen (BUN) level during the initial examination, diagnostic examinations and results showing the bleeding source, final diagnosis, and treatment.

The study protocol conforms to the ethical guidelines of the 1975 Declaration of Helsinki (6th revision, 2008) and was approved by the ethical review board of Saitama Children's Medical Center. Patient information was anonymized and collected, and an opportunity to withdraw participation was provided to the subjects and their guardians.

### Statistical Analysis

Descriptive statistics were used to describe the patient's demographic and clinical characteristics, endoscopic and other diagnostic test findings, and therapeutic procedures. Categorical variables were presented as percentages and numeric variables as means and ranges. Results were expressed as percentages or means ± standard deviation for continuous variables. The chi-square test, Fisher's exact test, and Mann–Whitney *U* test were used to compare non-continuous and continuous data. A univariate analysis of all patients was performed to identify the predictive factors of positive diagnosis via esophagogastroduodenoscopy (EGD). Then, a multivariate analysis of the predictive factors of positive diagnosis via EGD was conducted using the logistic regression model with odds ratio and 95% confidence interval (CI). *P* values of < 0.05 were considered statistically significant. Statistical analyses were performed using PRISM version 8.0 (GraphPad Software, San Diego, the USA) and EZR (Jichi Medical University, Saitama Medical Center, Saitama, Japan).

## Results

### Characteristics of Patients

**Figure 1:**
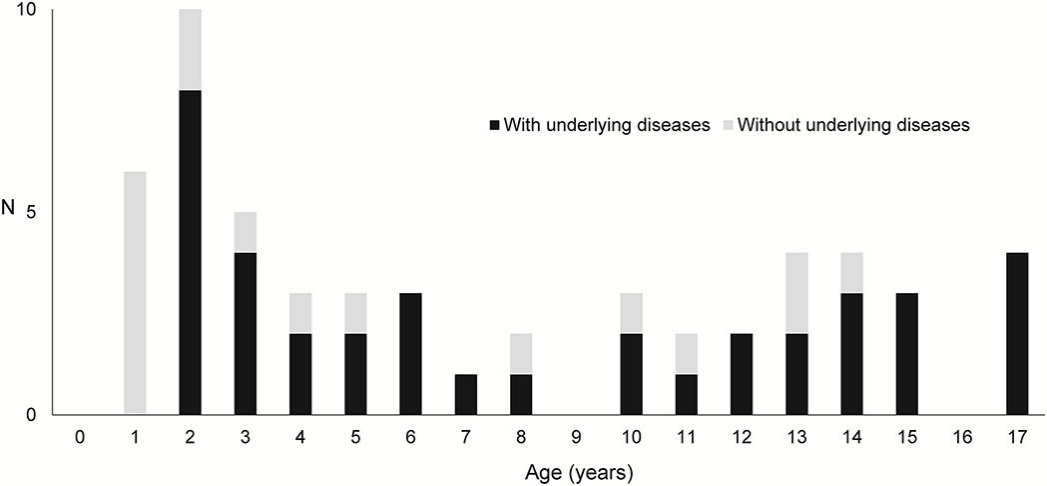
Age distribution and presence of underlying diseases.

Table 1 shows the case details. In total, 35 boys and 25 girls, with an average age of 7.8 years, were enrolled in this study. Then, 38 patients had underlying diseases such as malignancy and severe mental and physical disorders. Figure 1 depicts a graph of age and underlying disease. The age distribution was characterized by two peaks, which were as follows: one for children aged 1 and 2 years and another for those aged 10 years and above. Patients with underlying diseases were distributed across all ages. However, all patients aged 1 year old had no underlying diseases. The mean Hb (g/dL), Ht (%), and BUN (mg/dL) levels during the initial examination were, 9.2 ± 2.8, 28.2 ± 7.9, 17.5 ± 12.4, respectively. In total, 37 patients presented with accompanying symptoms such as fever, vomiting, abdominal pain, and diarrhea. Moreover, 26 patients received blood transfusion in addition to the specific treatment for diagnosed diseases. Only one patient whose source of bleeding could not be identified required blood transfusion.

**Table 1:** Characteristics of patients.

Sex (male:female ratio)	35:20
Age, months (range)	93.9 ± 65.5 (12–213)
Height, cm (range)	115.6 ± 30.7 (71–173.7)
Weight, kg (range)	24.1 ± 15.4 (6–57)
Underlying disease	
Malignant disease	10
Allergic disease	9
Chromosome disorder	6
Chronic liver disease	4
Chronic cardiac disease	2
Severe physical disability	2
Others	5
None	17
Accompanying symptom	
Abdominal pain	24
Vomiting (Bloody or tarry vomiting)	20 (9)
Fever	12
Diarrhea	9
Treatment	
Blood transfusion	26


### Diagnostic Examinations and Results

Table 2 shows the final diagnosis. The most common diagnosis was duodenal ulcer (*n* = 22), followed by small intestinal ulcer (*n* = 5), gastric ulcer (*n* = 4), esophagitis (*n* = 3), enteritis (*n* = 3), esophageal varices (*n* = 2), gastric tumor (*n* = 1), tongue bite (*n* = 1, later diagnosed as hemophilia B), gastritis (*n* = 1), and duodenitis (*n* = 1). The upper gastrointestinal tract was the most common source of bleeding (*n* = 34). In five patients, the source of bleeding was the small intestine. However, 16 patients did not present with abnormalities. Hence, the bleeding source could not be identified. In all cases of gastric and duodenal ulcer and gastritis and duodenitis, the presence of *Helicobacter pylori* (culture and histopathology) was assessed, and one patient tested positive for the bacteria. The causes of ulcers and gastroenteritis were identified in patients with eosinophilic gastrointestinal disease (*n* = 2), stasis-induced enteritis (*n* = 2), IgA vasculitis (*n* = 1), graft vs. host disease (*n* = 1, GVHD), drug (chemotherapy) (*n* = 1), inflammatory bowel disease-unclassified (*n* = 1, IBD-U), and adenovirus infection (*n* = 1).

**Table 2:** Final diagnosis (source of bleeding).

Duodenal ulcer	22
Small intestinal ulcers	5
Gastric ulcer	4
Esophagitis	3
Enteritis	3
Esophageal varices	2
Gastritis Gastric tumor Tongue bite (later diagnosed as hemophilia B)	1
No abnormal findings; hence, the bleeding source could not be identified	16

### Abdominal Ultrasonography

In total, 44 patients underwent abdominal ultrasonography (AUS). Moreover, abnormalities were found in 17 patients, of whom 15 presented with findings related to the final diagnosis. Thirteen patients showed thickening of the duodenal wall, and the final diagnosis in all of these cases was duodenal ulcer or duodenitis. In two cases of esophageal varices, the main portal vein could not be identified in the holus hepatis, and hence, cavernous transformation was suspected. Two patients showed thickening of the duodenal wall, but the bleeding source could not be identified.

### Esophagogastroduodenoscopy

In total, 55 patients underwent EGD. The mean and median time from the day of admission or consultation to EGD was 3.4 ± 9.2 days and 0 day, respectively. EGD was performed within 24 h in 30 cases and within 48 h in 40 cases. In 25 of 30 cases performed within 24 h and 35 of 40 cases performed within 48 h, the source of bleeding was identified. The most common finding was duodenal ulcer (*n* = 22), followed by esophageal varices (*n* = 6), esophagitis (*n* = 6), gastric ulcer (*n* = 4), gastritis and/or duodenitis (*n* = 4), gastric tumor (*n* = 1). Three patients with duodenal ulcers underwent endoscopic hemostasis. As shown in Table 3, the predictive factors of a positive diagnosis via EGD in the univariate analysis were Hb, Ht, and BUN levels, accompanying symptoms (vomiting and fever), abnormal AUS findings, time to referral for EGD, low Hb level, Hb level of ≤ 3 g/dL than the lower normal limit, but not age and underlying disease. Many of the causes of fever were not identified, but a viral infection was presumed as the cause. As shown in Table 4, the predictive factors of a positive diagnosis via endoscopy in the multivariate analysis were vomiting, abnormal AUS findings, and an Hb level of ≤ 3 g/dL than the lower normal limit.

**Table 3:** Predictive factors of a positive diagnosis via EGD in the univariate analysis.

	**EGD Dx-positive patients** **(*n* = 34)**	**EGD Dx-negative patients** **(*n* = 21)**	***P* value**
Sex	22:12	13:8	1
Age	96.8 ± 66.2	89.2 ± 64.0	0.55
Height	112.2 ± 33.6	117.1 ± 33.3	0.762
Weight	25.5 ± 19.5	25.8 ± 15.8	0.972
Hb level	8.2 ± 2.6	10.9 ± 2.4	0.00048
Ht level	24.9 ± 7.0	33.3 ± 6.4	0.000164
BUN level	21.2 ± 14.4	11.6 ± 3.1	0.00238
Accompanying symptoms			
Abdominal pain	16 (47%)	8 (38%)	0.584
Vomiting	19 (56%)	1 (4.8%)	0.00938
Bloody/tarry vomiting	9 (26%)	0 (0%)	0.00842
Fever	11 (32%)	1 (4.8%)	0.0194
Diarrhea	7 (21%)	2 (9.5%)	0.457
Underlying disease	20 (59%)	7 (33%)	0.0966
Positive findings on AUS	15 (26%; 58%)	2 (18%; 11%)	0.0209
Time to referral for EGD	0.73 ± 1.96	7.9 ± 13.5	0.0008
Low Hb level	−3.8 ± 2.8	−1.0 ± 2.6	0.000742
Low Hb level (≤ 3 g/dL than the lower normal limit)	24 (71%)	5 (24%)	0.00096

*EGD, esophagogastroduodenoscopy; Dx, diagnosis; AUS, abdominal ultrasonography*.

**Table 4:** Predictive factors of a positive diagnosis via EGD in the multivariate analysis.

	**Dx-positive patients (*n* = 34)**	**Dx-negative patients (*n* = 21)**	**Univariate analysis** ***P* value**	**Multivariate analysis**	
				***P* value**	**Odds ratio**
Vomiting	19 (56%)	1 (4.8%)	0.00938	0.00795	28.3 (2.4–333)
Positive findings on AUS	15 (26%; 58%)	2 (18%; 11%)	0.0209	0.01530	13.5 (1.65–110)
Low Hb level (≤ 3 g/dL the lower normal limit)	24 (71%)	5 (24%)	0.00096	0.04850	6.7 (1.01–44.3)

*EGD, esophagogastroduodenoscopy; Dx, diagnosis*.

### Small Bowel Capsule Endoscopy

In total, 20 patients, of whom five had confirmed patency using a patency capsule, underwent SBCE. The mean and median time from the day of EGD to SBCE was 2.6 ± 5.7 days and 0 day, respectively. Results revealed lesions in the small intestine in 12 patients. Further, four had findings that were relevant to the final diagnosis and treatment. Two patients had ulcers with active bleeding in the jejunum and the causative were GVHD and adenovirus infection. Two patients had an ulcer in the ileum and the causes were stasis-induced enteritis (solitary ulcer in a case of hypoganglinosis) and IBD-U.

### Other Modalities

Colonoscopy (CS) in 12 patients showed no significant findings in colon, although three patients had an ulcer in the terminal ileum. Twelve patients underwent Meckel's scintigraphy and no abnormal findings were reported.

## Discussion

This study first validated the pathogenesis of melena in children. Upper gastrointestinal lesions, including duodenal ulcers, accounted for 87% of all cases in which the bleeding source could be identified. EGD within 24 h is recommended for adults with melena and those suspected of upper gastrointestinal bleeding (6). This study showed that the same is true in children.

A common strategy for adults includes performing EGD and CS, followed by contrast-enhanced CT scan of the chest and abdomen if the bleeding source cannot be identified, is the first step in investigating gastrointestinal bleeding, including melena (7, 8). Next, SBCE or BAE is commonly performed. In this study, although CS did not identify lesions in the colon, three patients were found to have lesions in the terminal ileum, which could be diagnosed via SBCE. Although CS is widely performed on children, is safe (9), and may lead to a definitive diagnosis by biopsy, the rate of diagnosing a lesion in cases in which the colon is the bleeding source is low. In addition, in severe cases, bowel preparation is often not possible, making the identification of the source of bleeding difficult. Thus, with consideration of invasiveness, CS should be performed only when the bleeding source cannot be identified on EGD or SBCE. In addition, the likelihood of detecting neoplastic lesions via CT scan is low in children. Thus, contrast-enhanced CT scan is not effective in investigating gastrointestinal bleeding in children (10). In few cases, massive bleeding can be detected based on extravascular leakage of contrast media (11). Therefore, if prior examination shows no gastrointestinal stenosis and the bleeding source is not identified on EGD, SBCE could be performed using the capsule endoscope insertion device to identify the bleeding source in the small intestine, with consideration of invasiveness, in young infants or patients with dysphagia who cannot swallow. If the source of bleeding cannot be identified by endoscopy, angio-CT or Red cell scan should be considered.

If upper gastrointestinal bleeding is suspected, insertion of a nasogastric tube, aspiration, and saline lavage have been proposed (12). In addition, the immunohistochemical test of occult blood has a high sensitivity and specificity for the presence of gastrointestinal bleeding (13). However, the assessment of gastric contents with a nasogastric tube may induce vomiting and worsen the patient's condition, and AUS—a test to determine whether or not the stomach contents were bloody—can be performed. Thus, none of the previously mentioned procedures were performed in this study. AUS is a minimally invasive and simple examination and has a high detection rate for gastrointestinal lesions in children with a thin subcutaneous fat (14–16). By contrast, CT scan is less sensitive for diagnosing gastroduodenal ulcers (17). Hosokawa et al. showed that not only direct findings including thickening of the gastrointestinal wall and ulcers but also indirect findings such as hyperintense fatty tissue around ulcers and lymph nodes are useful in pediatric patients with gastric and duodenal ulcers (14). In this study, 15 of 17 children had abnormal AUS findings that were correlated with the final diagnosis. Ultrasonography is a simple, minimally invasive, and highly effective examination method. In contrast, the usefulness of AUS depends largely on the skill of the radiologist. AUS in patients with melena may be useful if performed by an experienced and skilled radiologist. The fecal occult blood test cannot be performed without defecation, and enema is not recommended for a patient with massive bleeding and poor general condition because it may cause deterioration. However, in patients with a stable general condition, it is reasonable to diagnose gastrointestinal bleeding by checking for the presence of fecal occult blood.

The use of SBCE has been approved in the United States in 2002 and in Japan in 2007. In the latter, the indication for pediatric use was expanded in 2010. SBCE has become an indispensable medical procedure in the current treatment of gastrointestinal diseases, the diagnosis of small intestinal diseases, and the evaluation of therapeutic outcomes even in children (18). The diseases identified via capsule endoscopy in children include ulcerative lesions such as those in Crohn's disease, Meckel's diverticulum, mass lesions such as juvenile polyps, and vascular malformation including angiodysplasia (19). SBCE is effective for diagnosing all these diseases. One of the complications of SBCE is retention, which occurs in 2.6% of patients with Crohn's disease and in 1.2% of those with obscure gastrointestinal bleeding (20). To prevent retention after capsule endoscopy, evaluation using a patency capsule is important in cases of suspected small bowel obstruction. This is especially essential in patients with suspected or diagnosed Crohn's disease. By contrast, the European Society of Gastrointestinal Endoscopy guidelines clearly state that the prior use of patency capsule is not required for obscure gastrointestinal bleeding (21). In addition, in younger children who cannot swallow or in patients with dysphagia, EGD guidance is required for the insertion of the capsule endoscope and patency capsule (22). In such cases, EGD must be performed twice, and its invasiveness should be considered. In this study, SBCE was useful in confirming the diagnosis in four cases. Nevertheless, the efficacy of SBCE for the diagnosis of small bowel bleeding in children can be confirmed by examining more cases in the future.

Finally, we assessed which tests that should be performed in specific patients and when to identify the bleeding source in children presenting with melena. Results showed that EGD should be performed within 24 h after hemodynamic stability is confirmed in patients with either a low Hb level or abnormal AUS findings. If the bleeding source from the esophagus to the duodenum is not evident, CS is generally performed as the second examination, but on the basis of the results of this study, we recommend SBCE as the second examination. CS should be considered if the bleeding source cannot be identified via EGD or SBCE. By contrast, if there is no significant decrease in Hb levels and AUS does not show any abnormality in the gastrointestinal tract, the presence of fecal occult blood should be evaluated. EGD should be considered in patients with a positive result in the fecal occult blood test. In the future, further validation including the method proposed in this study, is necessary in order to establish an optimal examination method for gastrointestinal bleeding specific to children, which is different from that for adults.

This study had two limitations. First the patients were from a single institution, and several had comorbidities. Therefore, the results might not reflect the condition of healthy children. Second, not all patients underwent the fecal occult blood test. Thus, in some patients, melena might not be attributed to gastrointestinal bleeding.

In conclusion, the upper gastrointestinal tract, particularly the duodenum, was the most common bleeding source in children with melena. As in adults, EGD is the primary endoscopic method of choice. Furthermore, SBCE may be useful for identifying the bleeding source in children without upper gastrointestinal lesions.

## Data Availability Statement

The raw data supporting the conclusions of this article will be made available by the authors, without undue reservation.

## Ethics Statement

The studies involving human participants were reviewed and approved by the Ethics Committee of Saitama Children's Medical Center. Written informed consent for participation was not provided by the participants' legal guardians/next of kin because: This study was retrospective and observational study, and an opportunity to withdraw participation was provided to the subjects and their guardians.

## Author Contributions

II conceived, designed, and drafted manuscript. All authors acquired, analyzed and interpreted the data, and approved the final version of the manuscript.

## Conflict of Interest

The authors declare that the research was conducted in the absence of any commercial or financial relationships that could be construed as a potential conflict of interest.

## Publisher's Note

All claims expressed in this article are solely those of the authors and do not necessarily represent those of their affiliated organizations, or those of the publisher, the editors and the reviewers. Any product that may be evaluated in this article, or claim that may be made by its manufacturer, is not guaranteed or endorsed by the publisher.

## Abbreviations

SBCE: small bowel capsule endoscopy
BAE: balloon-assisted enteroscopy
EGD: esophagogastroduodenoscopy
CI: confidence interval
GVHD: graft vs. host disease
IBD-U: inflammatory bowel disease-unclassified
AUS: abdominal ultrasonography
CS: colonoscopy.
